# Addressing gun violence across sectors in Durham, North Carolina

**DOI:** 10.3389/fpubh.2026.1820117

**Published:** 2026-06-11

**Authors:** Amy T. Fulton, Wanda Boone, Brian Eichner, Kate Elosta, Lorraine Graves, Krystal Harris, Uzuri Holder, Jackie Kaufman, John Moses, Kitty O'Hare, Jonathan D. Quick, Tricia Smar, Henry E. Rice

**Affiliations:** 1 Duke Global Health Institute, Duke University, Durham, NC, United States; 2 Together for Resilient Youth (TRY), Durham, NC, United States; 3 Duke University Hospital, Durham, NC, United States; 4 Duke Kunshan University, Kunshan, China; 5 Department of Social Work, North Carolina Central University, Durham, NC, United States; 6 Durham County, Department of Community Intervention and Support Services (CISS), Durham, NC, United States; 7 Violence Recovery Program, Duke University Hospital, Durham, NC, United States; 8 Durham County Firearm Injury Prevention Partnership, Durham, NC, United States

**Keywords:** community partnership, firearm injury and prevention, gun violence, harm reduction (HR), health equity, public health

## Abstract

Firearm-related death and injury are largely preventable. Coordinated efforts across sectors, including government, healthcare, and community partners, can substantially reduce firearm-related mortality and harm. Durham, North Carolina has a wide range of successfully implemented programs working to combat gun violence harm from many angles. Here, teams from several initiatives with partners across Duke University and the Durham community describe goals, outcomes, and challenges faced in their collaborations. These efforts represent multisectoral partnerships to improve support and resources for victims of gun violence, especially among racially marginalized individuals and communities. The landscapes shaping this issue and responses to it are explored, and individual partnerships are emphasized in shaping which initiatives are feasible. The incidence of gun violence is approached as a public health challenge. Together, these initiatives demonstrate a continued need for sustainable collaborations working to reduce inequities in gun violence. They emphasize the importance of grounding harm reduction efforts in trust and supporting prevention efforts with accessible resources. Successful interventions and programs are designed with significant involvement of the community, deliberate shared leadership, and early alignment of partnership goals. While local contexts will always shape this work, the successes of these programs may provide guidance for other communities working toward equitable firearm injury prevention and harm reduction strategies.

## Introduction

Firearm injury and deaths are preventable, and many interventions can lead to measurable reductions in harm. Evidence from the 2025 JAMA Firearms Summit Report indicates that coordinated efforts across government, healthcare, and community sectors can substantially reduce mortality related to gun violence. In select settings, state and local governments have implemented safety laws such as waiting periods, background checks, minimum age requirements, child access prevention laws, domestic-violence prohibitions, and extreme risk protection orders (1). Healthcare-based interventions have also shown success in different contexts, including lethal means counseling, safe storage guidance, hospital-based violence intervention programs, and structured safety planning (2–4). Community violence interruption, urban blight remediation, precision policing, and targeted social and economic supports have reduced shootings and homicides in some cities by more than half (5–8). Long-term analyses show that states and regions combining evidence-supported interventions for decades, such as California, New York, and Washington, DC, have achieved declines exceeding 40%, while states without sustained mitigation have seen substantial increases.

In Durham County, North Carolina, rates of firearm-related death have remained relatively constant over the past 20 years, with a slight increase from 12.5 deaths per 100,000 people in 2005, to 14.5 deaths per 100,000 people in 2025. The Black population remains disproportionately affected by gun violence, with 27.3 deaths per 100,000 in the last 10 years, compared to an overall population average of 14.7 in Durham County (9).

In this manuscript, teams from several Duke University and Duke Health organizations along with partners across the Durham community present collaborative approaches to addressing the challenges of gun violence and supporting victims of gun violence, especially among racially marginalized individuals and communities. We share descriptions and outcomes from programs across academic, government, and community partners.

## Firearm harm reduction in Durham, North Carolina

**Figure 1:**
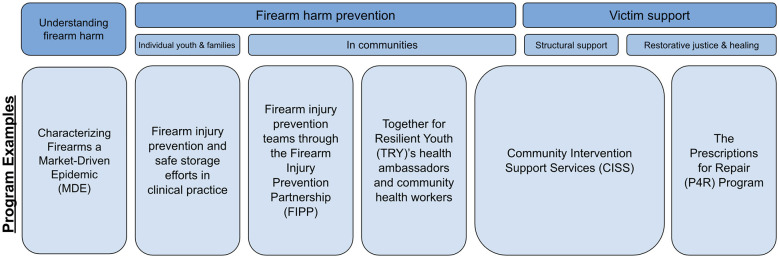
The Durham model of firearm harm reduction.

A variety of initiatives aim to address the needs of the Durham, North Carolina community. Supportive groups have developed methods of collaboration to bridge sectors in both preventing harm and supporting victims of harm. Programs within Durham work to address gun violence from many angles by understanding its incidence, preventing harm, and supporting victims (Figure 1). Descriptions of these collaborations, including goals, challenges, and outcomes, follow.

Throughout these descriptions, we refer to the “community” of all of Durham, North Carolina. However, from a health equity lens, we recognize the importance of multiple defined perspectives, especially from the groups most significantly impacted by gun violence. Specific groups—including racially marginalized groups—comprising communities within Durham are often the priority for the initiatives described. These efforts aim to understand and address the circumstances and needs of these groups within the broader community of all of Durham.

## Understanding firearms as a market-driven epidemic: implications for health equity

At the Duke Global Health Institute (DGHI), we have applied the market-driven epidemics (MDE) framework through interdisciplinary collaboration across academic, governmental, healthcare, and community sectors to examine how stakeholder dynamics, structural inequities, and policy environments shape firearm-related harm. This process usually requires translating complex systems concepts into approaches usable by community and policy partners and involves challenges common to firearm-related research, including political polarization, language sensitivities, and variation in state-level data that can limit analytic precision. Nevertheless, these experiences highlight the importance of shared vocabulary, transparent methods, and sustained communication across all aforementioned sectors.

The inequities in firearm injury observed in Durham are reflective of national patterns shaped by structural racism, economic marginalization, and many policy stakeholders. Framing firearms as an MDE provides a lens for understanding how these forces interact to produce predictable harms, particularly in racially marginalized communities.

Firearm injury in the United States follows social and structural gradients that shape unequal exposure to risk and access to protection. The firearms industry promotes gun ownership through fear-based messaging, predatory tactics, emphasis on lethality, suppression of research, and strategies used by other harmful-product industries (10). Evidence from classic MDEs such as tobacco and prescription opioids show that large-scale reductions in harm occur when governments, civil society, academia, and media coordinate effective interventions (10).

Our team at Duke has worked with national partners to examine how gun violence functions as a MDE (11). Firearm harm is concentrated in communities affected by structural racism, economic marginalization, and permissive policy environments. In 2021, state-level firearm death rates varied nearly tenfold, from 3.1 in Rhode Island to 29.6 in Mississippi (12). Firearm suicide, representing more than half of firearm deaths, disproportionately affects communities with economic stress and limited mental health access, with recent increases among Black youth (13). Women face greater femicide risk when a firearm is present (14), and firearm injury is now the leading cause of death among children and adolescents (15). These inequities reflect predictable outcomes of social and institutional conditions, with structural barriers limiting safe storage, prevention resources, and community protection (16). The MDE framework underscores how commercial and political forces drive unequal exposure and access to mitigation (10).

Narratives about firearms shape public understanding, policy, and perceptions of safety. Industry marketing and political advocacy have long framed firearms as essential for protection, autonomy, and identity while minimizing harm (5, 17). These narratives encourage perceptions of safety even when evidence shows that firearm access increases risks of suicide, assault, and intimate partner violence (14, 18, 19). This framing obscures the structural and commercial forces producing unequal harm and shifts attention toward individual behavior rather than policy conditions. Reframing firearms as an MDE redirects focus to commercial, political, and regulatory systems that shape risk exposure. The MDE lens clarifies how aggressive marketing, corporate resistance, limited research investment, and uneven policy environments disproportionately harm marginalized communities (10). A systems-level narrative also supports universal counseling approaches that reduce perceptions of profiling and strengthen trust.

The many actors which are involved in the MDE framework vary based on both their stance toward harm reduction and their power to enact change on this stance. Understanding variation in firearm mortality requires examining systems that shape policy feasibility and implementation. Various actors support, oppose, enable, or constrain firearm safety interventions, including government agencies, legislatures, civil society organizations, professional associations, industry, research institutions, survivor groups, and community organizations. The Power Support Matrix categorizes stakeholders by influence and policy position, revealing asymmetries in authority, resources, and agenda-setting. Integrating these dynamics with trend analyses clarifies which coalitions facilitate or impede equitable mitigation.

We have operationalized the MDE framework by mapping stakeholder configurations that shape firearm-related policy adoption, implementation, resistance, and long-term mortality trajectories (Table 1). Consistent with the MDE emphasis on the “who” and “why” underlying epidemic progression and mitigation, the matrix categorizes stakeholders by their relative power and orientation toward firearm safety interventions. This framework facilitates comparative examination of how stakeholder orientation among governmental, legal, healthcare, advocacy, industry, and community actors may influence the feasibility of sustainability of firearm harm-reduction strategies.

**Table 1:** Stakeholder power-support matrix.

	Supportive	Neutral	Opposed
High power	• Federal agencies (CDC, DOJ, ATF) • Certain state governments (e.g., CA, NY, DC) • U.S. surgeon general • U.S. congress (select members, post-2019 funding restoration)	• City governments in politically divided/swing states • Some federal courts interpreting PLCAA narrow exceptions	• Gun industry (manufacturers, distributors) • NRA • Gun rights absolutist legislators • Firearm lobby groups
Medium power	• Civil society advocacy organizations (everytown, giffords, march for our lives, brady, global action on gun violence) • Professional associations (AMA, APA, ABA) • Research institutions (e.g., RAND, Yale School of PH, Johns Hopkins, DGHI, Duke Center for Firearms Law)	• Large retailers (e.g., Walmart, Dick's) who have partially withdrawn from sales • Regional gun violence research coalitions	• Certain state governments (e.g., MO, MS, NM) • State legislators repealing safety laws • Lobby-aligned “think tanks”
Low power	• Public health practitioners • Survivors and victim advocacy groups • Hospital-based intervention programs (CVI, HVIP, C2G) • Urban blight remediation projects	• Healthcare workers not engaged in policy • Passive firearm owners • Local NGOs with no policy leverage	• Individual gun owners who strongly oppose regulation • Online communities (via social media) promoting anti-regulatory rhetoric

This power-support typology supports our examination of stakeholder configurations as core elements of mitigation or stagnation in the firearm market-driven epidemic.

Understanding the stakeholder landscape and structural landscape is important to framing the work of Durham-based initiatives. Both national and local policy contexts shape the development of these initiatives. The power, influence, and support configurations of stakeholders in the community invariably influence the needs and feasibility of prevention efforts, as described in the following sections.

## Firearm injury prevention in clinical practice

Although the contribution of injury from firearms to morbidity and mortality, particularly in children and adolescents, is increasingly recognized by health care professionals, prevention remains under-emphasized in health care settings. Gun violence prevention was part of curricula at only 13%−18% of American medical schools between 2015 and 2018 (20). It is logical to include these discussions in preventative health care visits; pediatricians regularly counsel patients about topics such as car safety, drowning prevention, and sun protection (21), and thus can introduce this topic from a trusted voice on the subject of child safety.

The majority of health care professionals do not routinely discuss safe firearm storage with patients and their families—only 7.5% of firearm owners reported having ever discussed this with a provider (22). Although most pediatricians agree that safe storage discussions are their responsibility, the majority of pediatricians provide this guidance at fewer than half of well-child visits (23). Similarly, only 25% of adult medicine physicians in North Carolina discuss firearm safety “often” or “very often.” (24) Common barriers for pediatricians to discuss firearm injury prevention include limited time, not enough resources, broaching a topic deemed as political, and fear of offending families. However, the majority of gun owners surveyed *do* believe that safe firearm storage is an appropriate conversation with their physician (25).

Part of this dynamic includes the perception of messaging coming from academia. It is hard to take what is seen on the news (such as extended coverage of mass shootings) and apply it to home life, where many keep a firearm for protection. Consistent outreach to improve family education of the risk of gun ownership is required, particularly on the increased rates of suicide when a firearm is present in the home (26).

Our medical center has worked with the Durham County Health Department's Firearm Injury Prevention Program to train health workers in discussions about gun violence prevention. Health care providers from Duke provide seminars to our trainees and others on how to have these discussions and why they are relevant. These discussions are based on the experiences from the Duke Children's Primary Care Gun Safety Initiative that started in 2006, with recent extension throughout Duke's primary care network.

Taking a “universal precautions” approach to discussions of safe storage with all patients and families allows providers to say “we have this conversation with everyone,” rather than risk profiling specific demographics. In other areas such as research recruitment, leveraging information from electronic medical records (EMRs) to initiate discussion has increased equity in research participation (27). Whether this is true for firearm injury prevention remains to be seen. It is now standard care in Duke Children's Primary Care to ask about the presence of guns in the home and to discuss safe gun storage and offer free gun locks. This effort to intercede in the threat of gun violence is aimed at all patients, regardless of their background.

Despite some initial hesitancy on the part of clinicians to incorporate gun violence prevention in pediatric care, it is now an accepted component of our practice and is folded into other anticipatory safety-related conversations such as poisoning prevention and the use of car seats. It is rare to encounter push-back from parents when gun safety is discussed. Rather, most parents appreciate conversations about gun violence prevention. In a 2015 survey, 75% of parents (including 71% of gun-owning parents) believed that pediatricians should counsel about safe storage (28). Patients and parents often offer compelling stories of how gun violence has affected their lives personally. When it is disclosed that a family has suffered from gun violence, an opening is created to offer support services and to begin to formulate gun violence prevention strategies going forward.

The collaborative approach to preventing gun violence began in 2005, thanks to a partnership with the Durham County Health Department Gun Safety Team, who have facilitated ongoing access to free gun locks. Additional partnerships include other clinical sites within the Duke Health System including the Duke Hospital Pediatric Emergency Room. Interest in gun violence prevention has grown in recent years as seen in the formation of the Duke Gun Violence Prevention Committee with members drawn from throughout the university and health system. Community organizations, in particular North Carolinians Against Gun Violence, have also been critical in engagement with local and national political activity around gun-related legislation.

## The Durham County firearm injury prevention partnership training program

In 2022, with support from the North Carolina Department of Health and Human Services (NCDHHS) Injury Prevention Branch, the Durham County Firearm Injury Prevention Partnership (FIPP; formerly Gun Safety Team) launched a five-part training series based on the Durham County model. The goal was to assist counties across North Carolina in establishing firearm injury prevention teams within their communities. Participants represented a range of sectors, including healthcare providers, emergency medical services personnel, hospital staff, public health departments, violence prevention organizations, suicide prevention groups, veterans' organizations, community educators, and other community-based organizations, many of whom serve historically marginalized populations.

The curriculum included a segment led by Duke Health providers, who shared insights from their Safe Storage Initiative and experience integrating safe firearm storage counseling into clinical practice in partnership with community-based firearm injury prevention efforts. This collaboration enabled providers to connect with populations beyond traditional healthcare settings. Attendees of the training shared the information within their own communities, leveraging their trusted roles to broaden the message's reach. By working together, these partners reached communities that no single organization could engage on its own.

To date, 23 counties have completed the training, including 13 rural, 6 urban, and 4 mixed counties, with a collective population of approximately 4.2 million people. This project demonstrates how collaboration between academic health systems, community organizations, and government can amplify firearm injury prevention efforts and serve as a model for implementation in diverse communities.

## Addressing challenges in cross-sector collaborations: the TRY program

To understand the challenges of cross-sector collaboration, it is critical to consider the perspectives of those directly impacted by gun violence. Recognizing the perspectives of the communities most affected and the impact of structural racial inequalities is essential. Insights from community based organizations and government representatives offer valuable reflections on these issues.

Together for Resilient Youth (TRY)'s work is informed by the socioecological model, which has shaped its efforts to reduce racial inequities in gun violence. Their prevention framework addresses risk and protective factors at individual, relationship, community, and systems levels, making it comprehensive and culturally responsive. In Durham's most vulnerable neighborhoods, TRY's health ambassadors and community health workers provide peer-to-peer support, trauma-informed engagement, and maintain a consistent presence. These strategies succeed because they are deeply rooted in proximity, trust, and the lived experiences of the communities most affected by violence.

From a community leadership perspective, interventions are most effective when they respect community wisdom and prioritize relationship building. Meaningful impact is observed when prevention strategies are co-created with residents who understand the historical and structural inequities shaping their lives. This approach is vital for addressing racial disparities in gun violence, as authentic solutions must originate from those who experience the greatest inequity.

Community partners often feel that challenges in collaboration don't originate in the communities, but rather in the policies from institutions. Although many grassroots organizations often show readiness and capacity for partnership, academic and governmental entities often hesitate to engage in genuine, power-sharing partnerships. This reluctance slows progress and sustains the very inequities that collaborative efforts seek to eliminate. Experience demonstrates that reducing racial inequality in gun violence requires more than implementing programs. It demands shared leadership and a commitment to aligning policy and practice with longstanding community knowledge about what works.

## Addressing challenges in cross-sector collaborations: the CISS program

As a government agency, the efforts of Community Intervention Support Services (CISS) to collaborate with academic and community partners to support victims of gun violence—especially those from racially marginalized groups—have revealed significant challenges and important lessons. Work with the Duke Violence Recovery Program and local organizations highlights the complexity of balancing different missions while responding to urgent needs. For example, a gunshot victim's immediate need may be relocation to prevent retaliation. The community partner organization may emphasize rapid, informal action to protect the victim's life; however, as a government entity, CISS must adhere to certain protocols and funding restrictions, while Duke is required to complete formal assessments and documentation before approving services.

A key challenge in gun violence collaborations is reconciling different operational priorities and limitations. Academic institutions often focus on research and evaluation, while community organizations emphasize immediate, culturally relevant services. Government agencies face policy constraints and funding limitations. These differences can lead to tension regarding timelines and expectations. Establishing shared goals early and maintaining transparent communication are essential for building trust and sustaining effective collaborations. These partnerships must collectively address the complex systemic challenges gun violence victims face, such as housing instability, employment discrimination, and limited access to mental health care—issues that disproportionately affect Black and Brown communities.

Community feedback underscores the importance of linking hospital-based advocacy with county-level social support, ensuring that interventions are not only evidence-based but also sustainable and grounded in lived experience. Ultimately, reducing racial disparities in gun violence relies on sustained, respectful collaborations that centers on the voices of those most affected and translates research into practice through policies driven by equity. These insights will guide the creation of comprehensive violence reduction programs that bring together science, practice, and policy to achieve lasting and equitable solutions for all communities.

## The prescriptions for repair program

**Figure 2:**
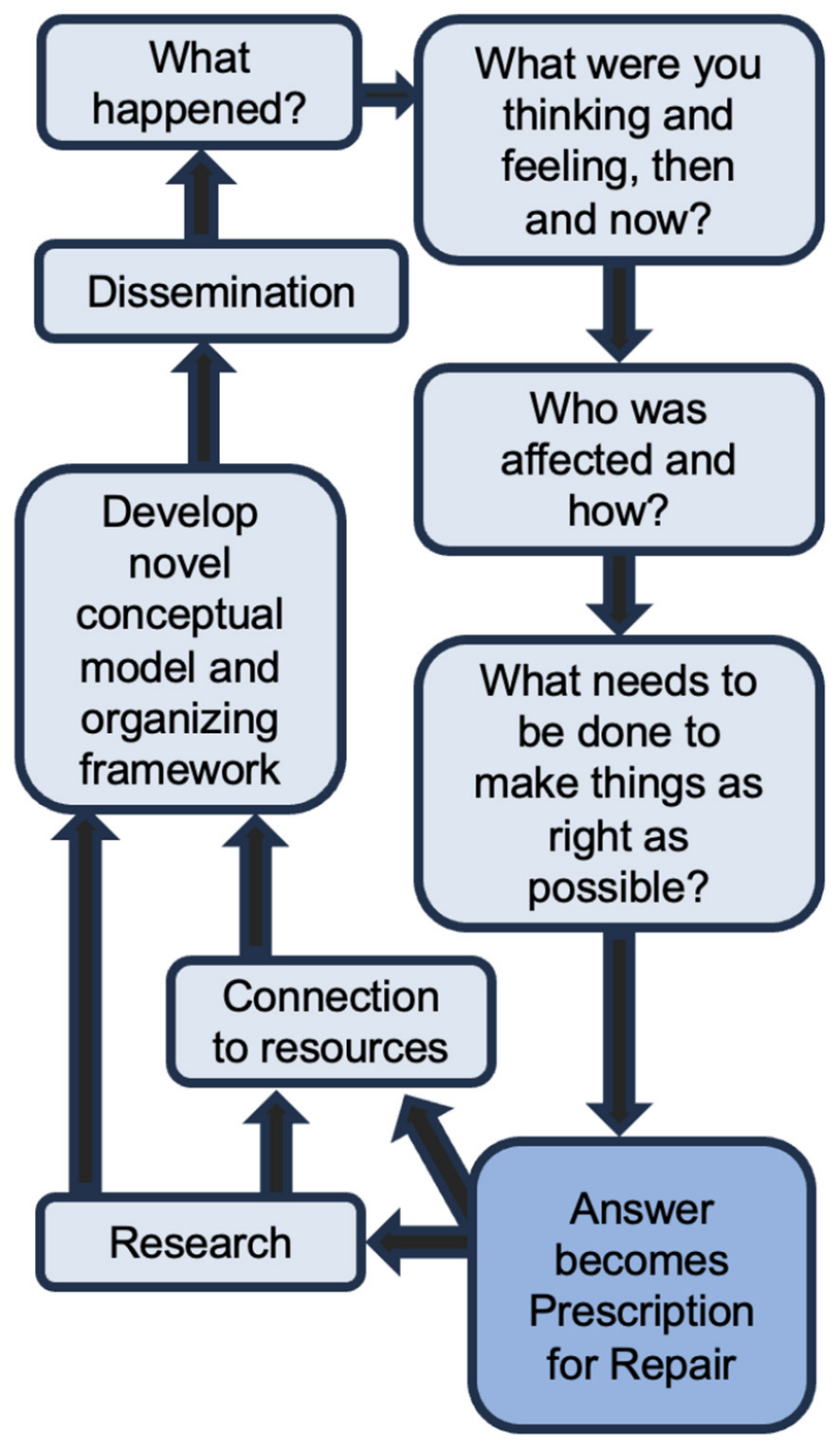
The prescriptions for repair listening session framework.

To be the target of intentional injury can have a profound impact on a person's sense of well-being. From Aug 2022 to Oct 2023, we operated a pilot program entitled Prescriptions for Repair (P4R) in Durham, which was supported by Durham community groups, government, and academic partners from Duke Health as well as North Carolina Central University (29, 30). Through a series of structured listening sessions using a restorative justice framework, trained community-based facilitators helped 30 participants (survivors of gun violence or loved ones of victims of gun violence) tell their stories through a non-judgmental narrative process (Figure 2).

Almost all participants expressed gratitude for the program. Although most facilitators were not licensed therapists, this program attests to the therapeutic and cost-effective nature of a listening program operated by trained lay community health workers for people impacted by gun violence (31). Note that this program was directed mainly toward survivors of community-based violence or their loved ones, not suicide survivors.

Most restorative justice programs are encounter-based, and focus on bringing together people experiencing conflict with those who commit violent action to address the harm created by the violence. From a restorative justice paradigm, changing the focus from reconciliation with perpetrators toward the victims of harm themselves allows for the opportunity for victims to express their thoughts regarding what happened and help victims feel heard. Many participants in P4R commented on the role of poverty, race, and racism in their personal experiences as well as with the challenges that local communities continue to face from gun violence. Participants confirmed the importance of structural disadvantages such as poverty and residential segregation in Black communities as underlying drivers of gun violence. This “community trauma” is characterized by a breakdown of social networks, relationships, and social norms across the community.

A program evaluation found that almost all participants in the P4R program felt that the listening sessions contributed at least somewhat to their personal healing, and many felt it would change the way they cared for themselves. Many participants also expressed that the program successfully gave them the opportunity to share their thoughts on breaking cycles of gun violence in Durham. Almost all respondents, including both participants and facilitators of the program, felt that P4R could contribute to reductions in gun violence.^33^ The evaluation also concluded that P4R supported the positive potential for impact from academic and institutional collaborations with community organizations. Specifically, the existing trusting community relationships maintained by community partners made the outreach and recruitment for this program successful (32). This kind of cooperation can promote short-term positive change and help lay the groundwork for long-term systemic shifts (32).

As P4R was a pilot program, the City of Durham recently opened an Office of Survivor Care (OSC) to continue community-based support programs for survivors of gun violence. The OSC is engaged with community, public, and academic groups across the region, working together to address the personal and community harm resulting from gun violence. As well, Durham City and County leaders recently launched a comprehensive Violence Reduction Plan with the goal of cutting homicides in half within seven years. This plan will be implemented in phases to identify strategies and to build engagement to reduce gun violence in collaboration across public schools, courts, academia, community groups, and social services.

## Discussion and lessons

These initiatives demonstrate the need for sustainable, cross-sector collaborations to reduce inequities in gun violence and address its occurrence as a public health challenge.

Across clinical, community, academic, and government contexts, several themes in addressing gun violence are clear. First, grounding harm reduction efforts in trust is essential, whether in the clinic or in the community. In some settings, such as clinical care, universal approaches and framing gun safety counseling as a part of standard preventative care efforts helps to reduce stigma, minimize potential perceptions of profiling, and support equity in delivery of interventions. However, it is also critical to understand the impact of structural harms and identify groups most significantly impacted by gun violence to design targeted programs.

Whenever possible, specific prevention efforts should also be supported by accessible resources, such as safe storage devices, established support groups, or clear referral pathways. This ensures initial discussions and intentions are linked to implementable goals and actions. Interventions and programs should be designed with significant involvement of the community, understanding of community wisdom and structure, and emphasis on community priorities.

When initiating partnerships across sectors, early alignment of partnership goals builds a foundation for successful collaboration. In multisector collaborations surrounding gun violence, consideration of frameworks such as the Market-Driven Epidemic can help define roles and priorities, and creates a shared vocabulary among stakeholders.

Considering these programs through the lens of the Power-Support Matrix, each initiative illustrates how actors with different structural authority and policy support can develop a strong partnership and collaborate successfully. We describe interventions across practice, county, city, and community levels, demonstrating how collaboration at each level reflects unique configurations of power and support. Although local implementation work will always reflect local context, it should also be considered through broader structural framing such as power and support, and the Market-Driven Epidemic.

The specific programs described here are grounded in the social, political, and institutional context of our setting in Durham, North Carolina. We hope the factors influencing their success may provide guidance for members of other communities seeking to design, implement, or scale equitable firearm injury prevention and harm reduction strategies.

## Data Availability

The original contributions presented in the study are included in the article/supplementary material, further inquiries can be directed to the corresponding author/s.
